# Exploring the Impact of Device Sourcing on Real-World Adherence and Cost Implications of Continuous Glucose Monitoring in Patients With Diabetes: Retrospective Claims Analysis

**DOI:** 10.2196/58832

**Published:** 2024-07-22

**Authors:** Jason C Allaire, Consuela Dennis, Arti Masturzo, Steven Wittlin

**Affiliations:** 1 Department of Psychology North Carolina State University Raleigh, NC United States; 2 Generativity Health Economics and Outcomes Research Chapel Hill, NC United States; 3 CCS Medical Clearwater, FL United States; 4 University of Rochester School of Medicine and Dentistry Rochester, NY United States

**Keywords:** diabetes, diabetic, adherence, medical costs, continuous glucose monitor, propensity score matching, CGM, glucose, cost, costs, claim, claims, insurance, economic, economics, finance, financial

## Abstract

**Background:**

Insurance benefit design influences whether individuals with diabetes who require a continuous glucose monitor (CGM) to provide real-time feedback on their blood glucose levels can obtain the CGM device from either a pharmacy or a durable medical equipment supplier. The impact of the acquisition channel on device adherence and health care costs has not been systematically evaluated.

**Objective:**

This study aims to compare the adherence rates for patients new to CGM therapy and the costs of care for individuals who obtained CGM devices from a pharmacy versus acquisition through a durable medical equipment supplier using retrospective claims analysis.

**Methods:**

Using the Mariner commercial claims database, individuals aged >18 years with documented diabetes and an initial CGM claim during the first quarter of 2021 (2021 Q1, index date) were identified. Patients had to maintain uninterrupted enrollment for a duration of 15 months but file no CGM claim during the 6 months preceding the index date. We used direct matching to establish comparable pharmacy and durable medical equipment cohorts. Outcomes included quarterly adherence, reinitiation, and costs for the period from 2021 Q1 to the third quarter of 2022 (2022 Q3). Between-cohort differences in adherence rates and reinitiation rates were analyzed using *z* tests, and cost differences were analyzed using 2-tailed *t* tests.

**Results:**

Direct matching was used to establish comparable pharmacy and durable medical equipment cohorts. A total of 2356 patients were identified, with 1178 in the pharmacy cohort and 1178 in the durable medical equipment cohorts. Although adherence declined over time in both cohorts, the durable medical equipment cohort exhibited significantly superior adherence compared to the pharmacy cohort at 6 months (pharmacy n=615, 52% and durable medical equipment n=761, 65%; *P*<.001), 9 months (pharmacy n=579, 49% and durable medical equipment cohorts n=714, 61%; *P*<.001), and 12 months (pharmacy 48% and durable medical equipment n=714, 59%; *P*<.001). Mean annual total medical costs for adherent patients in the pharmacy cohort were 53% higher than the durable medical equipment cohort (pharmacy US $10,635 and durable medical equipment US $6967; *P*<.001). In nonadherent patients, the durable medical equipment cohort exhibited a significantly higher rate of therapy reinitiation during the period compared to the pharmacy cohort (pharmacy 61/613, 10% and durable medical equipment 108/485, 22%; *P*<.001).

**Conclusions:**

The results from this real-world claims analysis demonstrate that, in a matched set, individuals who received their CGM through a durable medical equipment supplier were more adherent to their device. For individuals who experienced a lapse in therapy, those whose supplies were provided through the durable medical equipment channel were more likely to resume use after an interruption than those who received their supplies from a pharmacy. In the matched cohort analysis, those who received their CGM equipment through a durable medical equipment supplier demonstrated a lower total cost of care.

## Introduction

In 2021, an estimated 29.7 million people (8.9% of the US population) in the United States were living with diabetes [1]. Despite the availability of effective treatments, nearly half of all individuals with diabetes fail to achieve good glycemic control. According to US Centers for Disease Control and Prevention, an estimated 47.4% of adults with diabetes had a glycated hemoglobin (HbA_1c_) value of 7% or higher during the period of 2017-2020 [1], which is higher than the recommended HbA_1c_ goal of <7% for most nonpregnant adults with diabetes without significant hypoglycemia [2].

As a natural corollary of insufficient management, uncontrolled diabetes imposes substantial health consequences for patients in the form of cardiovascular complications, nephropathy, retinopathy, neuropathy, diabetic foot ulcers in advanced diabetes, and reproductive issues. Hyperglycemia has been associated with the spread of cancer cells, osteoarthritis, and an increased risk of infection [3]. These negative health outcomes impose a substantial burden on the health care system. In 2022, the estimated total direct and indirect costs of diabetes in the United States reached US $413 billion [4].

Managing diabetes involves consistent and ongoing care due to its chronic nature, and blood glucose monitoring has long been the gold standard for patients with diabetes to self-monitor their blood glucose levels for decades [5]. A successor to the familiar periodic fingerstick monitoring technique, continuous glucose monitoring enables individuals with diabetes to self-monitor their blood glucose continuously day and night, eliminating the burden of frequent, unpleasant finger pricks [5]. Continuous glucose monitors (CGMs) generate detailed reports that enable health care providers and individuals with diabetes to determine time in range, calculate glycemic management index, and evaluate hypoglycemia, hyperglycemia, and glycemic variability with certainty [6,7].

The effectiveness of CGMs is reflected in the 2023 American Diabetes Association Standards of Care, which included recommendations for using CGM in diabetes management [8]. Previous studies have shown that adherence to a CGM is significantly associated with reductions in HbA_1c_, medical costs, and health care use [9-12]. While CGMs have been a significant breakthrough in managing diabetes, work is needed to increase their use among the clinically appropriate population. The predictors of CGM adherence are well studied and include age, percentage of time in glucose target, the perceived necessity of CGM, BMI, and gender [13].

Another potential factor influencing adherence may be the dispensing source from which patients receive their CGM device. Depending on the benefits offered by a health plan, a physician’s prescription for CGM can be filled by a durable medical equipment supplier or a pharmacy. When a patient has a choice in dispensing source, the channel decision may be influenced by physician or patient preference, differences in patient out-of-pocket financial responsibility, or other factors.

No studies have been published examining the impact of the CGM device dispensing source on device adherence and costs, to the authors’ knowledge. To begin closing that knowledge gap, this retrospective analysis of insurance claims data assessed differences in adherence rates and costs among patients with diabetes obtaining CGM supplies through durable medical equipment providers and those using pharmacy services.

## Methods

### Data Source

Administrative claims data (January 1, 2021, to September 30, 2022) were obtained from the Mariner commercial claims database, which represents 75.7 billion claims of all payer types across 161 million unique patients across the United States.

### Population Analyzed

Patients with a diagnosis of type 1 or type 2 diabetes were identified using *International Classiﬁcation of Diseases, Ninth Revision* (249.00-250.99, 790.2, 790.21, 790.22, 790.29, 791.5, and 791.6) and *Tenth Revision* (E08.0 through E13.9) codes. Eligible patients were aged 18 years or older with an initial CGM claim in the first quarter of 2021, the exact date of which served as the index date. Patients with diagnosis codes for renal failure or cancer were excluded. Patients were required to have continuous enrollment for 6 months before and 15 months after their index date without evidence of CGM claims before the index date.

Two diabetes patient cohorts were identified by direct matching. The first cohort, the pharmacy cohort, was composed of patients who received their CGM device and subsequent supplies over the next 12 months through their pharmacy benefit. These patients were identified using the billing codes for the CGM devices and supplies. The second cohort, the durable medical equipment cohort, consisted of patients with diabetes who received their CGM device and supplies from a durable medical equipment provider over the same 12-month period. Patients in both cohorts were identified using the prespecified CGM and supply codes (Table S1 in the Multimedia Appendix 1). Patients from the durable medical equipment and pharmacy cohorts were matched directly based on Charleson Comorbidity Index scores, age range, gender, diabetes type, and insurance plan type.

### Outcome Measures

The 3 outcome measures were adherence, medical costs, and reinitiation.

### Adherence

Adherence was assessed after each patient’s index data at month 3, month 6, month 9, and month 12. These time points coincided with the prescribed 3-month ordering interval for CGM supplies. Patients were deemed adherent if they made all scheduled reorders, which served as a proxy for adherence. Any patient without evidence of a reorder during the study period was classified as nonadherent.

### Costs

Total medical costs, assessed throughout the 12-month follow-up period, included any medical or pharmacy claim reimbursed during the 12-month study period after each patient’s index date.

### Reinitiation

The reinitiation of CGM device use was assessed in any patient who became nonadherent during the 12-month study period. Reinitiation was defined as the resumption of CGM following a gap of ≥1 calendar quarter with no CGM codes occurring after a patient’s index date. Nonadherent patients were followed for 3 months after the 12-month assessment (15 months) to assess reinitiation in patients first showing nonadherence at 12 months. To be considered to have reinitiated CGM, the patient was required to resume the same type of device from the original device acquisition channel they had been using before the gap in therapy.

### Statistical Analysis

#### Cohort Assignment

Subjects were assigned to their respective cohorts by direct matching on the following matching variables: Charleson Comorbidity Index score (calculated using all existing claims for each patient over a 2-year period from the index date), age, gender, diabetes type, and insurance plan type.

#### Adherence Algorithm

The adherence algorithm uses the Medication Possession Ratio model, which is defined as the sum of the number of days supplied for all fills divided by the number of days in the given time. For the durable medical equipment cohort, supplies are assumed to be billed in a way that allows for a comparable analysis of prescription adherence.

More specifically, on the adherence calculations for both pharmacy and durable medical equipment cohorts, the patient must have at least 2 relevant claims; the numerator was the sum of units provided from all relevant claims; the denominator was calendar days from the chronologically first to the chronologically last claim in the time frame coded.

Differences in adherence and reinitiation rates between the durable medical equipment and pharmacy cohorts were examined using *z* tests, with the significance level set at *P*<.05.

#### Cost Analysis

Total medical costs included all allowable costs across pharmacy and medical benefits for patients with at least one medical claim in 2021 Q1. Pharmacy costs included all allowable costs for patients with at least one pharmacy claim in 2021 Q1. Outlier values for both total medical and pharmacy costs were removed by excluding data points that were 1.5 SDs above the average allowable cost for that service.

Differences in mean costs between the durable medical equipment and pharmacy cohorts were examined by 2-tailed *t* tests, with the statistical significance level set at *P*<.05.

### Ethical Considerations

Data were de-identified and comply with the patient requirements of the Health Insurance Portability and Accountability Act (HIPAA) of 1996; therefore, no review by an institutional review board was required per Title 45 of CFR, Part 46.101(b)(4) [14]. The authors obtained permission to use the data from PearDiver.

## Results

### Study Cohorts

**Figure 1:**
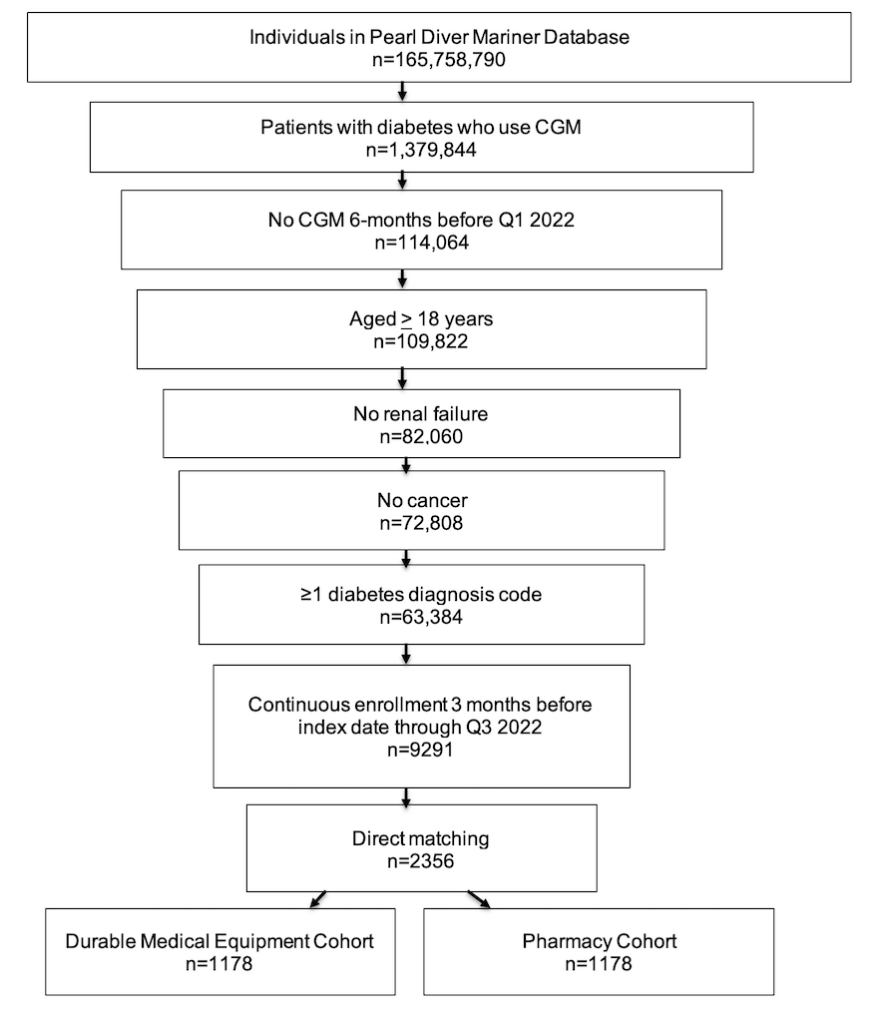
Sample selection.

Records for 165,758,790 individuals in the Mariner database were screened. Of these, 1,379,844 patients had diabetes and used CGM. After applying inclusion and exclusion criteria, 9291 patients (aged ≥18 years) with diabetes, an index CGM claim, no CGM claim in the 6 months before their index claim, and continuous enrollment 15 months after the index date were identified as individuals new to the use of CGM during the index period (Figure 1).

Direct matching generated two 1178 patient cohorts from these individuals. The first cohort, the pharmacy cohort, included patients who received their CGM device and subsequent supplies over the next 12 months through their pharmacy benefit.

The final study sample consisted of 2356 individuals with diabetes (pharmacy cohort=1778 and durable medical equipment cohort=1778) who were direct-matched and newly prescribed a CGM device. The mean age of both cohorts was 48.8 (SD 17.4) years. Patients’ baseline characteristics are presented in Table 1.

**Table 1:** Patient characteristics.

Characteristics		Durable medical equipment cohort (n=1178)	Pharmacy cohort (n=1178)	Total sample (N=2356)
Age (years), mean (SD)		48.9 (17.5)	48.7 (17.3)	48.8 (17.4)
**Gender, n (%)**				
	Man	591 (50.2)	591 (50.2)	1456 (49.7)
	Woman	587 (49.8)	587 (49.8)	1476 (50.3)
**Payer, n (%)**				
	Commercial	1106 (93.9)	1104 (93.7)	2210 (93.8)
	Medicare	27 (2.3)	27 (2.3)	54 (2.3)
	Medicaid	36 (3.1)	36 (3.1)	72 (3.1)
	Other or unspecified^a^	9 (0.8)	11 (0.9)	20 (0.8)
**Diabetes type**				
	Type 1, n (%)	760 (64.5)	760 (64.5)	1520 (64.5)
	Type 2, n (%)	132 (11.2)	132 (11.2)	264 (11.2)
	Other or unspecified^b^, n (%)	86 (24.3)	286 (24.3)	572 (24.3)
	CCI^c^, mean (SD)	1.19 (1.07)	1.19 (1.07)	1.21 (1.27)

^a^Other payers or payments include cash, employer groups, government, pharmacy benefit managers, processors, third-party administrators, or workers compensation.

^b^Others or unspecified may include diabetes of indeterminant etiology or rarer conditions, such as gestational diabetes mellitus, monogenic diabetes, or secondary diabetes.

^c^CCI: Charleson Comorbidity Index.

#### Adherence

The percentages of patients who were adherent within each quarter of the 12-month follow-up period are presented in Table 2. Adherence in the first 3 months was similar in the 2 cohorts. In both cohorts, adherence rates decreased over time; however, adherence rates were higher at 6, 9, and 12 months for the durable medical equipment cohort relative to the pharmacy cohort (*P*<.001).

**Table 2:** Adherence rate by diabetes cohort.

Time point, n (%)	Durable medical equipment cohort (n=1178)	Pharmacy cohort (n=1178)	*Z* score	*P* values
3 months	620 (52.6)	635 (53.9)	–0.06	.54
6 months	761 (64.6)	615 (52.2)	6.10^a^	.01
9 months	714 (60.6)	579 (49.2)	5.59^a^	.01
12 months	693 (58.8)	565 (48)	5.29^a^	.01

#### Health Care Costs

For adherent patients, the mean (SD) total allowable medical costs across the 12-month follow-up for the durable medical equipment cohort was US $6967 (SD US $5405). For the pharmacy cohort, it was US $10,635 (SD US $9095); the difference between the cohorts was statistically significant (t_1568.7_=–12.15; *P*<.001).

#### Reinitiation

In the durable medical equipment cohort, 22% (108/485) nonadherent patients resumed CGM, compared with 10% (61/613) nonadherent patients in the pharmacy cohort. The reinitiation rate was significantly higher in the durable medical equipment cohort (*z*=5.62; *P*<.001).

## Discussion

### Overview

Results of this retrospective insurance claims analysis indicate that patients who obtained their CGM device and supplies from a durable medical equipment cohorts supplier exhibited better adherence and incurred lower health care costs than patients who did so through a pharmacy. Despite a decline in adherence rates for both cohorts after the index CGM orders, adherence remained consistently higher in the durable medical equipment cohort than in the pharmacy cohort across subsequent assessments at 6 months, 9 months, and 12 months. The lower adherence seen in the durable medical equipment cohort at 3 months is the result of patients waiting longer for their second fill, resulting in an adherence lull at 3 months. The durable medical equipment cohort adherence rate increases at 6 months and aligns with expected patterns since most patients have gone through the refill process. Significant differences in medical costs accompanied differences in adherence between the durable medical equipment and pharmacy cohorts. For adherent patients, total medical costs were 53% higher in the pharmacy cohort relative to the durable medical equipment cohort.

Nonadherent patients were more likely to resume CGM if they received their device and supplies through a durable medical equipment supplier. After combining patients who were CGM adherent throughout the entire 12-month analysis period (durable medical equipment: n=693 and pharmacy: n=565) with those who resumed CGM after an interruption (durable medical equipment: n=108 and pharmacy: n=81), substantially more patients in the durable medical equipment cohort (801/1179, 68%) than in the pharmacy cohort (626/1179, 53.1%) were using their CGM device at the end of the analysis period. Although costs in patients who reinitiated CGM were not assessed, a higher overall rate of CGM use is likely to be accompanied by additional positive effects on resource use, but that remains a topic for future research.

While numerous analyses have described the positive impact of CGM on clinical outcomes and costs [10,15-17], as well as the severe negative consequences of nonadherence on costs [9,18,19], this analysis is the first to examine whether the distribution channel for CGM devices and supplies influences adherence and costs. A single study found that patients who received their CGM through their pharmacy had a faster time to initiate their CGM compared to patients who received their device through a durable medical equipment [20]. However, that study did not examine device adherence over time. Furthermore, no previous study has compared differences in costs between patients who received their CGM through pharmacy benefits or a durable medical equipment supplier.

Patients with diabetes not only face challenges associated with the correct use of CGM devices and CGM data interpretation but also frequently report psychological barriers to CGM use, such as not wanting to wear a device on their body, drawing unwanted attention, or losing privacy [21,22]. Parents of children with young children with type 1 diabetes report reluctance to use CGM devices due to painful insertions, problems with skin or adhesives, and the need to apply multiple devices to small bodies [22].

The difference in adherence between the 2 cohorts may be attributed to the extended services durable medical equipment suppliers provide. Durable medical equipment suppliers provide specialized support and personalized training on device usage, including initial setup, troubleshooting during ongoing use, and interpretation of data generated by the device. Durable medical equipment suppliers may also possess specialized expertise in specific disease states, such as diabetes, or have patient support staff capable of guiding clinicians and patients. This expertise allows them to promote increased patient awareness about CGM equipment and supplies, onboard new CGM users, explain subtle differences between CGM brands, discuss insurance benefits and medical policies specific to diabetes care, and address reorder objections. In contrast, while retail pharmacies can provide valuable information on multiple medications and supplies that a patient may be prescribed, they may not have the time or expertise to become experts in all aspects of care related to CGM devices and supplies or how to integrate CGM into a patient’s overall care plan, such as integrating CGM with insulin pump use. Finally, the high volume demands on pharmacy staffing may limit their ability to interact with patients or provide the ongoing equipment support a patient might need at home. Simply receiving a prescription can be a passive event, and it does not guarantee that the patient will receive the support needed to effectively use their CGM. With these services, patients ordering CGM directly from a durable medical equipment supplier may experience fewer disruptions in CGM and order CGM devices more consistently, potentially affecting adherence and costs.

Public insurance has very different rules for reimbursement relative to commercial insurance. Traditional Medicare only allows patients to access CGM from a durable medical equipment supplier, but Medicare Advantage plans frequently provide a choice between channels. When patients have a choice, improved outcomes and lower costs may encourage provision through a durable medical equipment supplier. Low-income households face additional challenges. All payers, especially state Medicaid agencies, have sought ways to manage expenses, and some have moved to provide CGM through the pharmacy channel to capture the rebates provided by manufacturers. If, as indicated by the current analysis, dispensing through a durable medical equipment supplier improves adherence and lowers costs, then obstacles to coverage for CGM in general and limiting distribution to pharmacies appear misguided.

The declining adherence over time observed in both cohorts is concerning and worthy of discussion. Strategies to improve adherence require a multifaceted approach that addresses both practical and psychological factors. These strategies should include patient education, personalized care, regular follow-ups, and addressing insurance coverage [21-24]. Providing education on the long-term benefits of consistent monitoring and CGM usage, including proper insertion techniques and data interpretation, can increase user confidence and comfort. Additionally, a personalized approach with regular follow-ups to set realistic goals, tailor the CGM regimen to their lifestyle, and provide feedback can motivate patients to stay on therapy. Lastly, insurance policies may dictate how patients can obtain their diabetes supplies, which often impacts patient cost-sharing, potentially creating financial barriers to adherence. The results of this analysis should prompt policy makers to advocate covering the cost of CGM devices and associated supplies to make them more accessible to patients from a source that promotes adherence. By implementing these strategies, patients can better manage their diabetes and avoid complications associated with poor adherence.

### Limitations

The results from this analysis should be considered alongside some caveats. First, while well-suited for evaluating health care resource use and costs, retrospective administrative claims data lack clinical detail, such as reasons for selecting a therapy, the brand or type of device and chosen sensors, and the specific clinical response. As a result, the analysis may not fully account for relevant clinical factors that contributed to outcomes. Second, the data for this study come from individuals with commercial health coverage or private Medicare supplemental coverage; therefore, the results of this analysis may not be generalizable to all CGM patients with other insurance or without health insurance coverage or to patients outside the United States. Third, because adherence was based on reorder rates, it is unknown whether patients used their devices correctly or at all.

Lastly, due to the nature of the claims data, it was not possible to determine why durable medical equipment patients had better adherence and lower costs. With respect to the latter caveat, it can be hypothesized that the reason for higher adherence among durable medical equipment patients centers around the operating business model durable medical equipment providers use, which is based on constant contact with the patient to obtain consent to ship or deliver equipment and supplies. Commercial payor durable medical equipment medical claim rules usually require consent to ship a CGM order. That, combined with the need to collect deductibles and coinsurance, results in a significant amount of patient contact. For commercial pharmacy refills, the automated process allows for quick refills that patients pick up or have delivered through a mail-order pharmacy. This nuance can result in faster device acquisition through a pharmacy, but followed by a progressive decline in adherence over time due to a lack of active patient engagement [20]. This may explain why the reinitiation rate in the durable medical equipment channel was significantly higher than in the pharmacy channel.

### Future Research

Future research should explore the potential impact of durable medical equipment supplier or patient interactions on psychological barriers to CGM. This would be an interesting area for future research. Nonetheless, previous research has shown a positive association between CGM uptake and patient education with a clinical diabetes educator [20]. In addition, studies should be conducted to evaluate adherence based on geographic differences in therapy availability and prescribing patterns. Additionally, geographic differences can influence device availability. Therefore, it is crucial to consider these factors while evaluating adherence.

### Conclusions

Results from this real-world, retrospective claims analysis demonstrate that greater patient adherence to CGM and lower health care costs significantly favor the acquisition of CGM devices and related supplies through a durable medical equipment provider instead of through a pharmacy. Given the effectiveness of CGM devices, the increasing prevalence of diabetes in the United States and worldwide, and the ever-shifting insurance landscape, further education of both providers and insurance plans is needed to ensure that patients receive and use CGM devices and supplies in the most cost-effective way.

## Appendix

Multimedia Appendix 1 Cohort and CPT and NDC Codes.

## Abbreviations

CGM: continuous glucose monitor
HbA1c: glycated hemoglobin
HIPAA: Health Insurance Portability and Accountability Act
