# Supplementary material for: Exploring the Impact of Device Sourcing on Real-World Adherence and Cost Implications of Continuous Glucose Monitoring in Patients With Diabetes: Retrospective Claims Analysis
Source: JMIR Diabetes. 2024 Jul 22;9:e58832. doi: 10.2196/58832 (PMC11301113; doi:10.2196/58832)
Supplement: Multimedia Appendix 1 [file diabetes_v9i1e58832_app1.docx]

**Table S1**. Cohort and CPT and NDC Codes.

| Cohort | CPT and NDC Codes |
| --- | --- |
| Durable medical equipment | CPT-A9278  CPT-K0553  CPT-K0554  CPT-A9277 |
| Pharmacy | NDC-76300000805  NDC-43169070405  NDC-63000017962  NDC-63000033698  NDC-63000035844  NDC-43169095568  NDC-63000028678  NDC-63000031699  NDC-63000035751  NDC-76300000260  NDC-63000028585  NDC-57599081800  NDC-08627001601  NDC-08627005303  NDC-08627700911  NDC-57599000101  NDC-57599000200  NDC-57599080000  NDC-57599080300 |
